# Peribronchial Arteriovenous Malformation with Cowden Syndrome: A Rare Case Report

**DOI:** 10.5761/atcs.cr.25-00171

**Published:** 2025-12-17

**Authors:** Shuta Sumitomo, Gouji Toyokawa, Yue Cong, Takatoshi Kubo, Hiroyuki Saigusa, Masaaki Sato

**Affiliations:** 1 Department of Thoracic Surgery, The University of Tokyo Hospital, Tokyo, Japan; 2 Department of Surgery and Science, Graduate School of Medical Sciences, Kyushu University, Fukuoka, Fukuoka, Japan; 3 Department of Radiology, The University of Tokyo Hospital, Tokyo, Japan; 4 Radiology Center, The University of Tokyo Hospital, Tokyo, Japan

**Keywords:** arteriovenous malformation (AVM), Cowden syndrome (CS), video-assisted thoracic surgery (VATS), racemose hemangioma

## Abstract

Cowden syndrome (CS) is a rare hereditary disorder caused by a germline variant of the *phosphatase and tensin homolog*, associated with multiple hamartomatous lesions occurring in various organs. Additionally, although rare, arteriovenous malformations (AVMs) with CS are found in the skin, brain, and spinal cord; however, peribronchial AVMs have not been previously reported. Herein, we report a rare case of a peribronchial AVM in a 30-year-old man with CS who presented with hemoptysis. Computed tomography (CT) revealed an AVM around the left upper bronchus, which was mainly fed by the left bronchial artery and drained into the left inferior pulmonary vein. Under video-assisted thoracic surgery, ligation of the feeding and draining vessels was performed. The AVM remarkably decreased in size one month after the surgery. This case highlights the need for whole-body contrast-enhanced CT to screen for AVMs and the importance of identifying feeding and draining vessels for optimal treatment methods.

## Introduction

Cowden syndrome (CS) is an autosomal dominant hereditary disorder caused by mutations in the *phosphatase and tensin homolog* (*PTEN*) gene, and it is estimated to occur at a frequency of 0.5 cases per 100000 population.^1)^ CS is known to be associated with multiple hamartomas of the skin, mucous membranes, brain, breast, thyroid, and colon. In some tissues, the hamartomas are associated with an increased risk of malignancy.^2)^ Cutaneous hemangiomas are recognized as occasional features of CS, but visceral arteriovenous malformations (AVMs) have been rarely reported; these are commonly observed in the skin, brain, and spinal cord.^2)^ To the best of our knowledge, there are no reports of peribronchial AVMs with CS. The current study presents a rare case of a peribronchial AVM with CS.

## Case Report

**Fig. 1:**
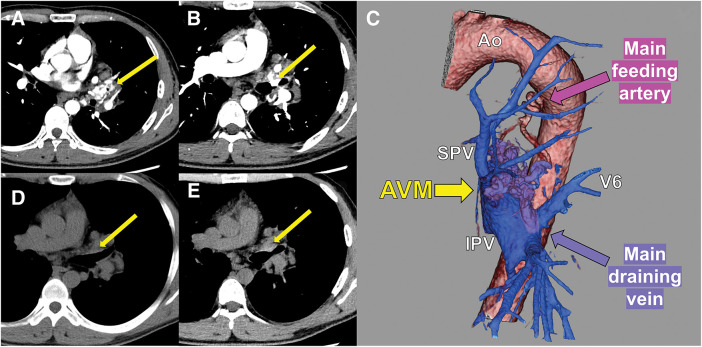
Findings of CT scans. (**A** and **B**) Contrast-enhanced CT on admission showing the AVM (yellow arrow) around the left upper lobe bronchus, which protruded into the bronchus. (**C**) 3D-CT showing the AVM (yellow arrow), which was mainly fed by the left bronchial artery (pink arrow) and drained (purple arrow) into the left IPV. (**D**) CT performed 4 years before surgery showing no mass (yellow arrow). (**E**) CT performed 1 year before surgery showing a small endobronchial mass (yellow arrow). Ao: aorta; AVM: arteriovenous malformation; CT: computed tomography; IPV: inferior pulmonary vein; SPV: superior pulmonary vein; 3D-CT: three-dimensional CT

We present the case of a 30-year-old male patient who presented with hemoptysis and chest discomfort. He had been clinically diagnosed with CS that was associated with esophageal glycogenic acanthosis, cervical lipomas extending into the mediastinum, adenomatous goiter, macrocephaly, and Lhermitte–Duclos disease, which is characterized by dysplastic cerebellar gangliocytoma. Genetic testing had not been performed in accordance with his wishes. The amount of preoperative hemoptysis per episode was less than 10 mL; however, it occurred about once a week, suggesting the need for medical intervention. No one among his first- and second-degree relatives had a definite history of CS; therefore, he was considered a sporadic case. Physical examination was unremarkable, and there was no relevant past medical history. Laboratory tests, including hemoglobin, were within normal limits. Contrast-enhanced computed tomography (CT) revealed an AVM around the left upper bronchus (**Fig. 1A**), which protruded into the bronchial lumen (**Fig. 1B**). Additionally, 3-dimensional CT (3D-CT) revealed that the AVM was mainly fed by the left bronchial artery originating from the descending aorta, and drained into the left inferior pulmonary vein (IPV) (**Fig. 1C**). A retrospective review of previous CT scans revealed that the AVM had gradually increased in size (**Figs. 1D** and **1E**).

**Fig. 2:**
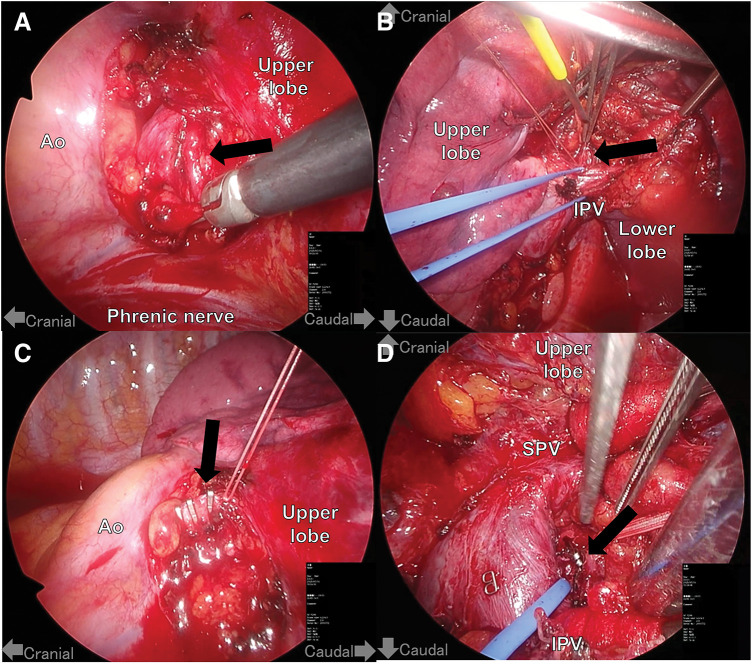
Intraoperative findings. (**A**) Main feeding artery (black arrow) and (**B**) main draining vein (black arrow) before clipping and ligation. (**C**) Main feeding artery (black arrow) and (**D**) main draining vein after clipping and ligation. Ao: aorta; IPV: inferior pulmonary vein; SPV: superior pulmonary vein

After discussion in a multidisciplinary conference, we decided to perform ligation of the main feeding artery and draining vein using video-assisted thoracic surgery (VATS). Although bronchial artery embolization (BAE) is an important treatment option for AVMs, we considered that it should not be performed in this case for the following reason: complete embolization of the AVM itself or feeding artery might not be achievable due to the tortuous nature of the AVM. This anatomical complexity might also be associated with an increased risk of procedure-related complications, such as cerebral infarction and bleeding during the procedure. He was placed in the right lateral decubitus position, and VATS was performed using four-ports. Under general anesthesia, he was intubated with a right-sided double-lumen endotracheal tube for lung deflation. The left bronchial artery, originating from the descending aorta, was found to be dilated and tortuous (**Fig. 2A**). As shown in the 3D-CT, the vein draining into the left IPV was also identified (**Fig. 2B**). Both the feeding artery and the draining vein were clipped with LigaClip and ligated with 2-0 silk (**Figs. 2C** and **2D**). The operative time was 5 hours and 21 minutes, with intraoperative blood loss of 200 mL.

**Fig. 3:**
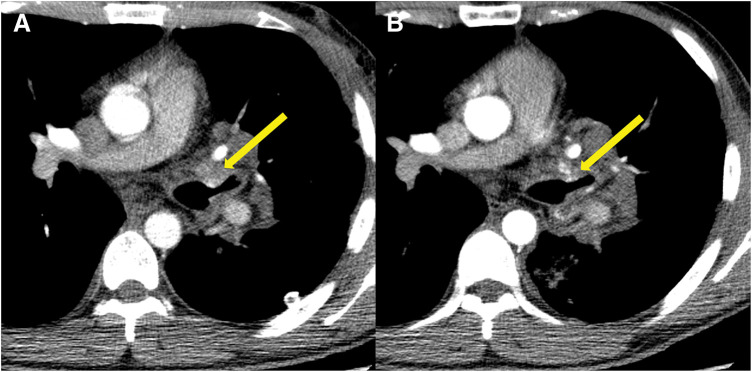
The shrinkage of the AVM on contrast-enhanced CT. CT showing the AVM (yellow arrow) on (**A**) postoperative day 2 and (**B**) day 30. AVM: arteriovenous malformation; CT: computed tomography

An enhanced CT on postoperative day 2 revealed a slight reduction in the size of the AVM (**Fig. 3A**). Left bronchial angiograms were also performed on postoperative day 4 (data not shown). They showed no contrast passage distal to the clips; however, the AVM was faintly stained via minor mediastinal feeders. Transcatheter arterial embolization was attempted, but it was unsuccessful due to severe vasospasm.

Postoperatively, the patient immediately experienced relief from chest discomfort. There were no postoperative complications, and the patient was discharged on postoperative day 6. A follow-up CT on postoperative day 30 revealed a significant reduction in the size of the AVM, along with its protrusion into the bronchial lumen (**Fig. 3B**). The patient is doing well approximately 4 months after surgery, without any episodes of hemoptysis and no regrowth of the AVM.

## Discussion

CS patients exhibit numerous multifocal vascular disorders, such as AVMs, hemangiomas, and hamartomatous vascular malformations.^3,4)^ The frequency of vascular disorders in patients with CS ranges from 18% to 34%, compared to 5% to 10% in the general population.^3)^ Vascular lesions in patients with CS are thought to result from the proteins encoded by *PTEN*, which regulates the function of vascular endothelial growth factor.^4)^ To date, 36 cases of AVMs associated with CS have been reported in the literature, as shown in **Table 1**, excluding frequently affected organs and tissues like the skin, mucosa, and soft tissues. AVMs associated with CS are most frequently found in the brain and spinal cord; however, there are no reports of peribronchial AVMs with CS similar to the current case.

**Table 1:** Locations and numbers of AVMs with CS reported in the literature (excluding skin, mucosa, and soft tissues)

Location	Intracerebral or spinal cord	Liver	Intrapelvic	Parotid gland	Heart	Intestines	Retina	Ovary	Vertebrae	Lower limbs
No. of cases	17	4	3	3	2	2	2	1	1	1

AVM: arteriovenous malformation; CS: Cowden syndrome

Peribronchial AVM, previously known as “bronchial artery racemose hemangioma,” is reportedly a rare vascular malformation.^5)^ The largest published series included 34 cases of peribronchial AVMs, with a right-sided predominance (25 cases) and no differences between males and females.^6)^ CT findings of peribronchial AVM include tortuous, dilated, and enlarged bronchial arteries, and in some cases, its protrusion into the bronchial lumen has also been reported, as in the present case.^7–9)^ These findings underscore the importance of contrast-enhanced CT when an AVM is suspected.

Contrast-enhanced CT and/or angiography are useful for identifying the feeding and draining vessels.^8)^ Treatment for peribronchial AVMs, including surgery and BAE, depends on the vascular anatomy, particularly the location, number, and diameter of the feeding and draining vessels.^5,9)^ Surgery comprises extirpation of AVMs, lung resection, including pneumonectomy, and ligation of vessels.^10–12)^ In this case, we performed surgery based on the following reasons: first, multiple vessels might be present and feeding arteries were tortuous, so catheter selection for all vessels was difficult; second, coils might cause proximal occlusion with no distal penetration into the AVM and preclude further endovascular access^13)^; third, a shunt in the AVM allowed embolic material to enter the systemic circulation and potentially cause embolic events in various organs, including the brain. Regarding the proximal ligation, the Japanese clinical guideline for interventional radiology (IVR) does not recommend proximal ligation or embolization for AVM.^14)^ However, in the present case, it was impossible to resect only the AVM due to its location around the left upper bronchus, and a left upper lobectomy with bronchoplasty, which would have been highly invasive, would have been required to completely remove the AVM. Given the invasiveness of the procedure and the patient’s age, we decided to perform proximal ligation. Additionally, because the draining vein emptied into the left atrium, backflow into the AVM might remain; therefore, distal ligation was also performed. However, we must carefully monitor the development of collaterals.

We successfully performed surgical treatment of the AVM, resulting in immediate relief from the chest discomfort. Postoperative contrast-enhanced CT showed a significant reduction in the size of the AVM. Despite this improvement, regular follow-up CT is necessary to monitor for any regrowth of the AVM after the surgery. In cases of recurrence, additional multimodal treatments, such as surgery and BAE, may be required.

## Conclusion

To the best of our knowledge, this is the first case report of a peribronchial AVM associated with CS. Full-body examinations, including contrast-enhanced CT, are important because vascular disorders can occur at multiple sites in patients with CS and may occasionally be life-threatening. Accurate identification of feeding and draining vessels can lead to the optimal treatment strategy.

## Declarations

### Data availability

The data supporting the results are provided within the article, and no further source data are needed.
